# Treatment of Advanced Metastatic Melanoma

**DOI:** 10.5826/dpc.11S1a164S

**Published:** 2021-07-01

**Authors:** Pietro Quaglino, Paolo Fava, Luca Tonella, Marco Rubatto, Simone Ribero, Maria Teresa Fierro

**Affiliations:** Dermatologic Clinic, University of Turin Medical School, Turin, Italy

**Keywords:** Metastatic melanoma treatment, anti-PD1, target therapy, metastatic melanoma survival, response rate

## Abstract

The introduction in clinical practice of new drug compounds both targeted therapies anti-BRAF and checkpoint inhibitors have largely improved our potential to manage advanced metastatic melanoma patients. This has led to a significant improvement in terms of response rates and particularly in the overall survival (OS). The long-term results of trials with follow-up data of patients treated with targeted or immunotherapies reported median OS rates around 24 months, with 5-year survival rates around 35-40%.

As to the drugs currently available and reimbursed by the Italian National Health System, 3 combinations of anti-BRAF/anti-MEK inhibitors are available (dabrafenib/trametinib, vemurafenib/cobimetinib and the most recently introduced encorafenib/binimetinib).

As for checkpoint inhibitors, first line immunotherapy is represented by anti-PD1 blockers (nivolumab and pembrolizumab), whilst the anti-CTLA-4 ipilimumab can be used as second line immunotherapy.

The decision-making factors that define the best treatment approach in stage IV patients with metastatic melanoma include the mutation pattern, performance status, high/low tumor load, brain metastases, progression pattern (low/fast), and availability of clinical trials.

This review will analyze the current therapeutic tools adopted for the treatment of metastatic melanoma patients. It will then focus on the latest results obtained by novel treatments (checkpoint inhibitors and targeted therapies) which can be used in the clinical daily practice.

## Introduction

The presence of distant metastases with soft tissues or internal organs’ involvement is classified as stage IV metastatic melanoma. According to the 8th edition American Joint Committee on Cancer (AJCC) 2018, M1 is defined by both the anatomic site of distant metastatic disease (“a” for soft tissue, “b” for lung, “c” for other sites, and the new “d” designation added to include distant metastasis to the central nervous system), and serum lactate dehydrogenase (LDH) values (designated as “0” for not elevated and “1” for elevated [1]. Only a minority of melanoma patients develop distant metastases during the course of their disease, thanks to early diagnosis.

Even if the prognosis for stage IV patients is still severe, the introduction of the new drug compounds, both targeted therapies anti-BRAF and checkpoint inhibitors, have largely improved our potential to manage these patients inducing a significant improvement in terms of response rates (RR) and particularly overall survival (OS). The unsatisfactory results obtained by (bio)-chemotherapy were clearly summarized in a review study by Korn et al [2], which analyzed clinical data obtained from more than 2,000 patients enrolled since 1975 in 42 phase II trials. An overall 1-year survival rate of 25.5% and a median OS of 6.2 months were achieved, with no significant improvement during the last 30 years. The long-term results of the trials with follow-up data of patients treated with targeted or immunotherapies reported median OS rates around 24 months, with 5-year survival rates around 35–40% [3].

Currently, the main criterion adopted to decide the best therapeutic option for an advanced patient is the presence or absence of the BRAF gene mutation. The BRAF mutation is harbored by approximately 50% of melanomas. More frequently, those arising without chronic sun-induced damage, induce the hyperactivation of the MAP-kinase molecular cascade, leading to an uncontrolled proliferation of cancer cells [4]. In the presence of BRAF mutation, both anti-BRAF targeted therapies and checkpoint inhibitors can be used, whilst in the presence of a BRAF wild pattern, only immunotherapy can be prescribed [5]. As to the drugs currently available and reimbursed by the Italian National Health System, 3 combinations of anti-BRAF/anti-MEK inhibitors are available (dabrafenib/trametinib, vemurafenib/cobimetinib and the most recently introduced encorafenib/binimetinib). As to checkpoint inhibitors, first line immunotherapy is represented by anti-PD1 blockers (nivolumab and pembrolizumab), whilst the anti-CTLA-4 ipilimumab can be used as second line.

The decision-making factors defining the best treatment option in a stage IV metastatic melanoma patient are represented by: mutation pattern, performance status, high/low tumor load, brain metastases, progression pattern (low/fast), and availability of clinical trials.

This review will analyze the therapeutic tools available for the treatment of patients with metastatic melanoma and will focus on an update of results obtained by the new treatments (check point inhibitors and targeted therapies) which can be used in the clinical daily practice.

### Anti-BRAF and Anti-MEK Inhibitors

The pharmacological inhibition of the mitogen-activated protein kinases (MAPK) pathway by targeting the mutant v-Raf murine sarcoma viral oncogene homolog B1 (BRAF) is a milestone in the management of metastatic melanoma.

2 randomized phase III studies highlighted the efficacy of the dabrafenib-trametinib combination as first-line treatment in metastatic melanoma: COMBI-d (n=423, comparing the combination of dabrafenib/trametinib versus dabrafenib) and COMBI-v (n=704, which compared the combination versus vemurafenib). The primary endpoint was progression-free survival for the COMBI-d trial and overall survival for COMBI-v. In the COMBI-d study, the RR was 69% for the dabrafenib-trametinib combination and 53% for dabrafenib alone (p=0.0014). The median PFS was 11 months for the dabrafenib-trametinib combination and 8.8 months for dabrafenib monotherapy (p=0.0004); the median OS was 25.1 months and 18.7 months, respectively (p=0.01) [6]. Furthermore, the dabrafenib-trametinib combination had a better safety profile and improved health-related quality of life as well as reducing pain [6, 7]. As for the COMBI-v, the objective RR was 64% in the dabrafenib-trametinib combination arm and 51% in the vemurafenib arm (p<0.001) [8]. The dabrafenib-trametinib combination significantly improved OS compared to vemurafenib monotherapy (26.1 compared to 17.8 months). Median PFS in dabrafenib–trametinib arm was 12.1 months and 7.3 months in the vemurafenib arm [8]. In agreement with COMBI-d, the side effects’ analysis and quality of life in the COMBI-v trial favored the combination over the monotherapy [8,9].

The results of the pooled analysis of the 2 trials were evaluated after a 22 month-follow-up period, on 563 patients in total, with 5-year PFS of 19% and 5-year OS of 34%. Complete responses occurred in 19% of patients and were associated with an improved long-term outcome, with an OS rate of 71% at 5 years. In multivariate analysis, several baseline factors (eg performance status, age, sex, number of organ sites with metastasis, and lactate dehydrogenase levels) were significantly associated with both progression-free survival (PFS) and OS [10]. In particular, in a 3-year landmark pooled analysis, baseline LDH level and number of organ sites were confirmed to be significantly associated with PFS and OS. In addition, baseline sum of lesion diameters (SLD) was identified as a predictor for progression. In the most favorable prognostic group (normal LDH, SLD <66 mm, <3 organ sites), 3-year PFS was 42% [11].

Despite significant improvements with the combination therapy, BRAF/MEK inhibition is still frequently complicated by acquired resistance, most often via upregulation of MAPK signaling. In the clinical management, one possible therapeutic strategy is represented by the treatment beyond progression. This is defined as the continuation of targeted therapy in responding patients who showed an isolated disease progression that can be treated by a loco-regional approach. Retrospective data suggest that treatment with BRAF inhibitors beyond progression is associated with improved survival, even if prospective data are still needed [12,13].

Besides clinical trials, compassionate-use programs provided a relevant opportunity to retrospectively evaluate the treatment patterns and clinical outcomes in a real-world setting and to validate the results derived from controlled randomized clinical trials. In particular, DESCRIBE III was a large international multicenter study, which enrolled 509 patients. Patients were categorized into 3 groups based on their observed treatment duration: long-term (on therapy ≥12 months), intermediate (on therapy ≥6 months and <12 months), and short-term (on therapy <6 months) duration of benefit. In agreement with the results of the pooled analysis of COMBI-d and COMBI-v, normal LDH level and <3 metastatic sites at baseline, were associated with a longer duration of treatment benefit in a real-world setting [14].

Also, the combination of vemurafenib and cobimetinib had a better outcome than vemurafenib alone in a phase 3 randomized clinical trial performed on 495 patients with previously untreated, unresectable, locally advanced, or metastatic BRAF V600 mutation-positive melanoma (coBRIM). The combination showed a significantly higher clinical activity in terms of RR (68% vs 45%), PFS (median: 9.9 vs 6.2 months), and survival (9 months OS 81% vs 73%) [15]. At a median 14.2-month follow-up, the median PFS was 12.3 months for the combination versus 7.2 months for placebo and vemurafenib (p<0.0001). Median OS was 22.3 months for cobimetinib and vemurafenib versus 17.4 months (for placebo and vemurafenib; p=0.005). The safety profile for cobimetinib and vemurafenib was tolerable and manageable, and no new safety signals were observed with longer follow-ups [16].

The clinical activity of a third combination schedule of anti-BRAF/anti-MEK was investigated in the clinical trial COLUMBUS. COLUMBUS was a 2-part, randomised, open-label, phase III study. During part 1, patients were randomly assigned (1:1:1) to receive oral encorafenib 450 mg once daily, plus oral binimetinib 45 mg twice daily (encorafenib plus binimetinib group), oral encorafenib 300 mg once daily (encorafenib group), or oral vemurafenib 960 mg twice daily (vemurafenib group). Part 2 of the study compared encorafenib 300 mg once daily plus binimetinib 45 mg twice daily with encorafenib 300 mg once daily alone. At 3-year analysis, median OS was 33.6 months with encorafenib plus binimetinib and 16.9 months with vemurafenib [17, 18]. Median PFS was 14.9 months in the encorafenib plus binimetinib group and 7.3 months in the vemurafenib group. A confirmed overall response by blinded independent central review occurred in 63% of patients in the encorafenib plus binimetinib group compared with 51% in the encorafenib group, and 40% in the vemurafenib group. The median time to response was 1.8 months for the encorafenib plus binimetinib group. The most common grade 3–4 adverse events seen in more than 5% of patients in the encorafenib plus binimetinib group were increased γ-glutamyltransferase (9%), increased creatine phosphokinase (7%), and hypertension (6%).

### Anti-CTLA4

Ipilimumab is a fully humanized monoclonal antibody that binds to CTLA-4, a receptor expressed on the T-cell surface that interacts with CD80 (B7-1) and CD86 (B7-2) on the Antigen-Presenting-Cells (APCs) and downregulates T-cell response. CTLA-4 blockade allows CD28 to bind to B7-1 receptors, leading to immune activation, IL-2 secretion, cytotoxic T-cells expansion, and proliferation [19]. The interaction between CTLA-4 and B7-1/2 takes place in an early phase of the immune response, involving “naive” T lymphocytes and the APCs. This mechanism of action explains the characteristics of the clinical activity as well as the common side effects of this drug, consisting of immune-mediated reactions (irAEs) developing more frequently in the skin, gastro-intestinal tract (mainly diarrhea), liver and endocrinal glands). The trial that led to registration of ipilimumab in melanoma was a phase III trial in which ipilimumab ± glycoprotein 100 peptide (gp100) vaccine was compared with gp100 vaccine monotherapy in patients with unresectable stage III or stage IV melanoma. Ipilimumab monotherapy significantly improved median OS compared with gp100 vaccine monotherapy (10.1 months vs. 6.4 months) [20]. In another important randomized phase III trial, the combination of ipilimumab (10 mg/kg) and dacarbazine (850 mg/sqm) resulted in significantly superior OS compared to dacarbazine (850 mg/sqm) plus placebo (11.2 months vs. 9.1 months) [21].

Ipilimumab produced a plateau in the survival curves: a recent pooled analysis of OS data for 1.861 patients enrolled in 10 prospective and 2 retrospective trials, with up to 10-year follow-up, showed that the survival curve began to plateau around 3 years after treatment. 3-year OS rates were 22%, 26%, and 20% for all, treatment-naive, and previously treated patients, respectively [22]. Moreover, the results of the ipilimumab expanded access programme (EAP) in Italy resulted consistent with these data, confirming the activity of the drug also in specific patient’s subsets such as the elderly, the mucosal or uveal primaries, and in the presence of brain metastases [23].

### Anti-PD1

PD-1 represents a co-inhibitory receptor involved in the negative regulation of T-cell activation [24]. The expression of PD-1 ligand (PD-L1) on tumor cells induces the development of an immunosuppressing environment through the ligand with the PD-1 expressed on T lymphocytes, thus leading to T-cell inhibition and cancer immune system escape.

Two anti-PD-1 monoclonal antibodies are available in the clinical practice and can be used for the treatment of metastatic melanoma patients, ie nivolumab and pembrolizumab.

The CheckMate 066 trial investigated nivolumab monotherapy as first-line treatment for patients with previously untreated BRAF wild-type advanced melanoma. In this multicenter, double-blind, phase III study, 418 patients with previously untreated, unresectable, stage III/IV, wild-type BRAF melanoma were randomly assigned 1:1 to receive nivolumab or dacarbazine, with OS as primary endpoint. The results demonstrated superior overall RR (40% vs. 13.9%, respectively) and increased 1-year OS (72.9% vs. 42.1%, respectively). Moreover, nivolumab treatment-related adverse events occurred in 11.7% of the patients receiving nivolumab and 17.6% of the patients receiving dacarbazine, respectively [25]. At 5-year analysis [26], ORR was 42% with nivolumab and 14% with dacarbazine. Five-year OS rates were 39% with nivolumab and 17% with dacarbazine; PFS rates were 28% and 3%, respectively. Among patients treated with nivolumab who had a complete response (20%), 88% (37 of 42) were alive as of the 5-year analysis.

In CheckMate 037 phase III trial, patients were randomly assigned 2:1 to receive nivolumab 3 mg/kg every 2 weeks or investigators’ choice chemotherapy (ICC) in ipilimumab-refractory patients with advanced melanoma [27]. Primary endpoints were the proportion of patients who had an objective response and OS. At first interim analysis on 120 and 47 randomized patients, confirmed objective responses were reported in 31.7% of patients in the nivolumab group vs. 10.6% of patients in the ICC group; no treatment-related deaths occurred. In the final 2018 report [28], the overall RR (27% v 10%) and median duration of response (32 versus 13 months) were significantly higher for nivolumab versus ICC. Fewer grade 3 and 4 treatment-related adverse events were observed in patients on nivolumab (14% v 34%). Median OS was 16 months for nivolumab versus 14 months for ICC; this data should however be interpreted with caution as patients enrolled in the ICC group could thereafter be treated by anti-PD1 or anti-BRAF targeted therapies.

As to pembrolizumab, KEYNOTE-006 was an open-label, multicenter, randomized, controlled, phase 3 study in which 834 patients with advanced melanoma were randomized to receive pembrolizumab at a dose of 10 mg/kg every 2 or every 3 weeks, or with 4 doses of ipilimumab (3 mg/kg every 3 weeks). The estimated 6-months PFS rates were 47.3% for pembrolizumab every 2 weeks, 46.4% for pembrolizumab every 3 weeks, and 26.5% for ipilimumab, respectively. Estimated 1-year OS rates were 74.1%, 68.4%, and 58.2%, respectively. The RR was improved when pembrolizumab was administered either every 2 or 3 weeks, as compared with ipilimumab. Treatment-related adverse events of grade 3–5 severity were lower in the pembrolizumab groups (13.3% and 10.1%) [29]. At the final 5-year follow-up data, median overall survival was 32.7 months in the combined pembrolizumab groups and 15.9 months in the ipilimumab group (p=0·00049). Median PFS was 8.4 months and 3.4 months, respectively [30]. A relevant analysis from this study was done in patients who stopped pembrolizumab after 24 months as per protocol. After a median follow-up of 34·2 months from completion of pembrolizumab, the estimated 24-month PFS from treatment interruption for all 103 patients was 78.4% and 36-month OS was 93.8%. Estimated 24-month PFS was 85.4% for patients with complete response, 82.3% for patients with partial response, and only 39.9% for patients with stable disease. These data pave the way for the possibility of interruption of anti-PD1 treatment in responding patients after 2 years of therapy.

KEYNOTE-002 study was a randomized phase II multicenter trial in which advanced melanoma patients with progression after ipilimumab and/or BRAF/MEK inhibitors were randomized to pembrolizumab 2 mg/kg or 10 mg/kg every 3 weeks or investigator-choice chemotherapy. Cross-over to pembrolizumab was allowed following progression on chemotherapy.

A total of 180 patients were randomized to pembrolizumab 2 mg/kg, 181 to pembrolizumab 10 mg/kg and 179 to chemotherapy. 6-month PFS was 34% in the pembrolizumab 2 mg/kg group, 38% in the 10 mg/kg group, and 16% in the chemotherapy group [31]. At the final post-hoc 5-year analysis, the ORR was 22% and 28% in patients receiving pembrolizumab, versus 4% in patients receiving chemotherapy (p<0.0001 for both pembrolizumab doses versus chemotherapy) [32].

### Anti-CTLA-4/anti-PD-1 Combo Regimens

Preclinical models have shown that double inhibition of CTLA-4 and PD-1, when compared with single-molecule inhibition alone, synergistically increases anticancer responses 173.

In the double-blind phase II CheckMate 069 study, patients were randomized to treatment with ipilimumab + nivolumab or with ipilimumab + placebo. At a median follow-up time of 24.5 months, the two-year survival was 63.8% for patients treated with nivolumab and ipilimumab in combination and 53.6% for patients treated with ipilimumab alone. In patients with wild type BRAF melanoma, the RR was 61% in the group of patients who received combination therapy compared to 11% of patients who received ipilimumab + placebo (P <0.001), with complete responses. reported in 22% of patients in the first group and none in patients treated with ipilimumab alone [33].

The Phase III CheckMate 067 [34,35] study assigned patients with previously untreated advanced melanoma to receive one of the following regimens: nivolumab (at a dose of 1 mg per kilogram of body weight) plus ipilimumab (3 mg per kilogram) every 3 weeks for 4 doses, followed by nivolumab (3 mg per kilogram every 2 weeks); nivolumab (3 mg per kilogram every 2 weeks) plus ipilimumab-matched placebo; or ipilimumab (3 mg per kilogram every 3 weeks for four doses) plus nivolumab-matched placebo. The 2 primary end points were PFS and OS in the nivolumab-plus-ipilimumab group and in the nivolumab group, as compared with the ipilimumab group. 945 patients with advanced melanoma not treated with previous therapies were recruited. Combination therapy showed significantly higher PFS (11.5 months, 95% CI 8.9–16.7) than nivolumab monotherapy (6.9 months, 95% CI 4.3–9.5), or ipilimumab (2.9 months, 95% CI 2.8–3.4). The risk of death or tumor progression was reduced by 58% compared with ipilimumab monotherapy (HR 0.42; 99.5% CI 0.31–0.57). The ORR was 57.6% (95% CI, 52.0–63.2) in the combination cohort versus 43.7% in nivolumab (95% CI, 38.1–49.3) and 19% (95% CI, 14.9–23.8) in the ipilimumab monotherapy group. Patients treated with the combination therapy showed a complete response in 11.5% (compared with 8.9% with nivolumab and 2.2% with ipilimumab monotherapy). Most interestingly, when patients were stratified for PD-L1 negativity or immunohistochemical staining positivity (less or more than 5% of PD-L1 stained tumor cells in a section of at least 100 tumor cells), the median PFS was 14.0 months for patients with PD-L1 positive tumors in both the nivolumab-plus-ipilimumab group, and the nivolumab group. In contrast, in patients with PD-L1-negative tumors, PFS was longer with combination therapy than with nivolumab alone (11.2 months [95% CI, 8.0 a not achieved] vs. 5.3 months [95% CI, 2.8 to 7.1]). In this study, nivolumab combination therapy was superior to nivolumab monotherapy or ipilimumab alone in patients with PD-L1 negative tumors, whereas in patients with PD-L1 positive tumors there was no significant difference between nivolumab monotherapy and combined therapy. Overall survival at 5 years was 52% in the nivolumab-plus-ipilimumab group and 44% in the nivolumab group, as compared with 26% in the ipilimumab group. No sustained deterioration of health-related quality of life was observed during or after treatment with nivolumab plus ipilimumab or with nivolumab alone [36]. Grade 3 or 4 treatment-related adverse events occurred in 59%, 23%, and 28% of the patients in the nivolumab-plus-ipilimumab, nivolumab, and ipilimumab groups, respectively.

This combination therapy was approved in Europe by the EMA on May 2016, regardless of patients’ PD-L1 status.

### cKIT Inhibitors

Mutations and amplification of the KIT oncogene are more frequent in melanomas arising in the skin with chronic sun damage, acral sites, or mucosal melanomas. A number of evidences from laboratory analysis and preclinical studies showed that hot-spot mutations, most frequently constituted by substitutions at exons 11 and 13, induce a pathological activation of the KIT, thus an upregulation of the downstream signal transduction pathways, which are not only the MAP-kinase, but also the PI3K/AKT, and JAK/STAT pathways. KIT gene expression has been correlated with activating mutations, which indicates the role of KIT in tumorigenesis in melanoma. Therefore, KIT has been suggested to be a potential therapeutic target for malignant melanoma.

Several trials have been conducted using KIT-targeted tyrosine kinase inhibitors in melanoma in both selected and unselected patient populations. Trials with imatinib showed responses if KIT was mutated but not if it was wild-type and amplified [37–39]. Other KIT inhibitors such as dasatinib, sunitinib, and nilotininb have also exhibited responses in KIT-mutant melanomas. However, taken together, these studies showed a percentage of responses around 20% and 30%, mostly of short duration without a significant impact on survival. Moreover, all these studies were performed on a relatively small number of patients and there is no available randomized trial. In a recent retrospective analysis of 78 patients with metastatic melanoma harboring c-Kit mutations or amplifications treated with imatinib, ORR and DCR were 21.8% and 60.3%, respectively. The median OS and PFS of all patients were 13.1 [40]. The limited clinical activity of targeting cKIT imply that cKIT mutant patients should be treated as first line with immune check point inhibitors and only after the failure of these regimens, consider the potential of cKIT inhibitors.

### NRAS Mutant Patients

NRAS mutations (codons 12, 13, and 61) can be detected in 15–20 % of all melanomas. These alterations have been associated with aggressive clinical behavior and a poor prognosis; however, a recent retrospective multicenter Italian study did not confirm the unfavorable prognostic significance of NRAS mutation. A cohort of 331 patients treated with immunotherapy as first-line were retrospectively recruited: 162 NRAS-mutant/BRAF wild-type (mut/wt) and 169 wt/wt. Regarding the outcomes, no significant differences were reported in overall RR, PFS or OS. Irrespectively of the mutational status, a longer OS was significantly associated with normal LDH, <3 metastatic sites, lower white blood cell and platelet count, lower neutrophil-to-lymphocyte (N/L) ratio [41].

Some studies have been reported analyzing the clinical activity of anti-MEK inhibitors in these patients. Based on these data, a randomized phase III trial was designed, comparing binimetinib with dacarbazine. The study enrolled 269 patients in the binimetinib arm and 133 in the dacarbazine arm. Binimetinib significantly prolonged PFS and improved RR with respect to the control arm even if the clinical benefit is slow, with median PFS of 2.8 months compared to 1.5. Furthermore, no differences in OS were achieved. An interesting point was that the benefit in terms of PFS appear to be higher in patients with a prior immunotherapy (median 5.5 months) even if this is a retrospective analysis and thus caution should be taken [42].

### Patients with Brain Metastases

The presence of brain metastases is now classified as stage IV M1d and it is associated with a poor prognosis (median survival 4 months) [43, 44] Patients with active brain metastases are in fact in most cases excluded from phase III clinical trials, particularly those involving immunotherapies [45,46]

As to targeted therapies, the COMBI-MB was a multicenter, multicohort, open-label, phase 2 study evaluating the combination of dabrafenib/trametinib in 4 patient cohorts with melanoma brain metastases (based on the presence of symptoms, ECOG, and previous radiotherapy). Percentages of intracranial responses ranged from 44% to 59% with PFS lower than that found in patients with no brain metastases (19% PFS at 12 months). Dabrafenib plus trametinib was active with a manageable safety profile in this melanoma population that was consistent with previous dabrafenib plus trametinib studies in patients with BRAFV600-mutant melanoma without brain metastases, but the median duration of response was relatively short [47].

In phase II studies that involved the use of nivolumab or pembrolizumab alone in patients with brain metastases, the percentage of responses varied from 16% to 25%, that is clearly lower than the standard immunotherapy RR of around 40%–50%. The duration of the responses was also significantly shorter [45].

The results of 3 studies carried out in patients with active brain metastases have instead highlighted the clinical activity of the combination of anti-PD1 nivolumab with ipilimumab in these patients with RR ranging from 46% to 55%.

In particular, the ABC study is a phase 2 study that randomized patients with asymptomatic brain metastases to receive the combination nivolumab + ipilimumab, versus nivolumab alone. A third arm involved the inclusion of patients with symptomatic brain metastases to receive exclusively nivolumab. Intracranial RR were 51% in patients treated with the combination, 20% in asymptomatic patients treated with nivolumab, and 6% in symptomatic patients treated with nivolumab. The rate of intracranial responses increased to 59% with the combination in naive, non-pretreated patients, compared to 21% with monotherapy. PFS was also significantly different, 43% at 3 years with the combination versus 15% and 6% with monotherapy, respectively. The safety profile did not report significant differences with respect to that highlighted in previous studies of immune combo with a percentage of adverse events grade ¾ higher than monotherapy but still manageable from a clinical point of view in a patient setting with such a severe prognosis as those included in the study [48].

A second study reporting the results of the ipi nivo combination in patients with brain metastases is the Phase II Check-Mate 204 study, which enrolled 75 patients, with 55% intracranial and 53% global RR, 2.8 months’ time to response and median duration of responses not yet achieved [49].

The third NIBIT-M2 study is a randomized phase 3 study that included patients with brain metastases randomizing them into three arms (fotemustine, fotemnustine + ipilimumab, and nivolumab + ipilimumab). The arm treated with the immune combo obtained 44% intracranial response with PFS 36% at 4 years and 41% OS at 4 years [50].

### New Scenarios: Combining Targeted and Immunotherapies

A novel approach which is emerging for BRAF-mutant patients is represented by the combination of targeted therapies and immune-checkpoint inhibitors [51], commonly referred as “triplets”. The rationale for this association is 2-fold. From the clinical point of view, it could couple the principal benefits of the 2 regimens, thus the high response rate of the targeted therapies and the remission duration of immunotherapies in an attempt to overcome the development of acquired resistance. From the biological point of view, anti-BRAF targeted therapies have been recognized to positively modulate the immune regulation, by promoting T-cell infiltration with reduction of regulatory T-cells, inducing melanoma antigen-expression, and restoring the impaired MHC-I surface expression, thus reducing the immunosuppression and immune escape associated with the BRAF mutated oncogenic pathway [52].

The KEYNOTE-022 trial [53], the first phase II trial investigating a triplet in melanoma, randomized 120 patients to receive dabrafenib/trametinib plus pembrolizumab or placebo, with PFS as primary endpoint. The study did not show a statistically significant difference in PFS, even if a non-statistically longer PFS was found in patients treated with the triplet (16.9 vs 10.7 months at 36 months follow-up); moreover, median duration of response was 25.1 months in the triplet cohort and 12.1 months in the control group. Patients treated with the triplet experienced however higher toxicity rates, with 58.3% developing grade 3–5 treatment-related adverse event versus 26.7%.

The IMspire150 trial was a randomized phase 3 study comparing the triplet atezolizumab, vemurafenib, and cobimetinib versus vemurafenib, cobimetinib and placebo, with the primary endpoint of PFS. A total of 514 patients were enrolled. At a median 18.9 month follow-up, investigator–assessed PFS was significantly longer in the triplet group versus control (15.1 vs 10.6 months; p=0.025). The triplet was approved by FDA; however, even if the study met its primary endpoint, the values of the median PFS reached is similar to that of the Keynote-022. The frequency of grade 3–4 adverse events was similar (79% versus 73%); no major adverse events were found in the triplet group and the percentage of patients who stopped all treatment due to adverse events was similar (13% in the triplet versus 16%) [54].

More recently, the results of the part 3 of the COMBI-I trial were presented [55]. This phase III randomized clinical trial enrolled 532 patients to compare the combination of dabrafenib and trametinib plus spartalizumab or placebo. The PFS was longer in the triplet group even if the difference did not meet a statistical significance (16.2 versus 12 months). The objective response rate was 69% versus 64%. The percentage of patients showing grade 3 or more treatment related side effects was 55% in the triplet group versus 33% in the control arm.

The results of the large randomized trials comparing the triplets versus the standard targeted regimens did not completely confirm thus until now the promising preliminary data, also showing a less favorable toxicity profile for this associations. However, all the studies identified a longer PFS of the triplet versus the control arm (with a statistical significance only in the IMspire trial but with similar values across the different studies), thus it is justified to wait for a longer follow-up time to better characterize the role of the combination of targeted and immunotherapies, and to identify which could be the patients that could benefit more from this treatment.[Fig f1-dp11s1a164s][Fig f2-dp11s1a164s][Fig f3-dp11s1a164s][Fig f4-dp11s1a164s]

## Conclusions

The comparison between OS rates before the development of new drugs (1-year survival 25%, median survival 6 months) [2] and those achieved with both targeted therapies and immune check point inhibitors (5-year survival 35%, median survival 24 months) clearly highlights the relevant impact that these new treatment approaches are having in the disease course of advanced metastatic melanoma and this is well recognized by the main Italian national, European and American guidelines [5,56,57]. However, when considering the curves from the other side, it is evident that at 5 years, 65% of patients die due to disease progression, supporting the need of more active treatment strategies or combinations. The results from the trials analyzing the clinical activity of the so-called triplets (combo-target plus anti-PD1) gave conflicting results and it is reasonable to think that more follow-up is needed. In the daily clinical practice, the challenges are represented by the management of patients with aggressive disease and/or multiple visceral sites, as well as those with brain metastases or mucosal/coroidal primaries. The availability of adjuvant treatments is improving the disease course in stage III patients disease-free after surgery but the management of the progressions occurring during adjuvant treatment, particularly in BRAF wild-type patients, still represents another clinical challenge. The availability of data coming from real life experiences together with the results of ongoing clinical trials will provide relevant informations to improve the management of these patients, as well as the identification of both prognostic and predictive factors associated with the disease course and response to treatment.

## Figures and Tables

**Figure 1 f1-dp11s1a164s:**
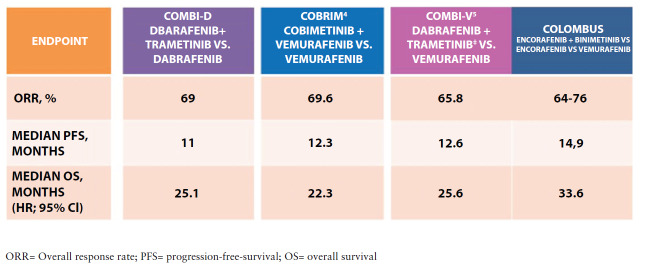
Clinical activity of the 3 main combinations of targeted therapy. ORR= Overall response rate; PFS= progression-free-survival; OS= overall survival

**Figure 2 f2-dp11s1a164s:**
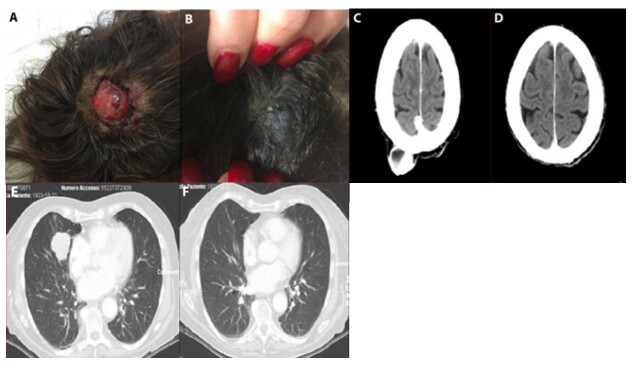
(A, B) Development of response in a representative patient with BRAF mutant metastatic melanoma with lung and skin metastases: response achieved as clinically evident at the 7th week from the beginning of treatment. (C–F) CT scan performed 3 months after the beginning showing the complete clearance of lung metastases

**Figure 3 f3-dp11s1a164s:**
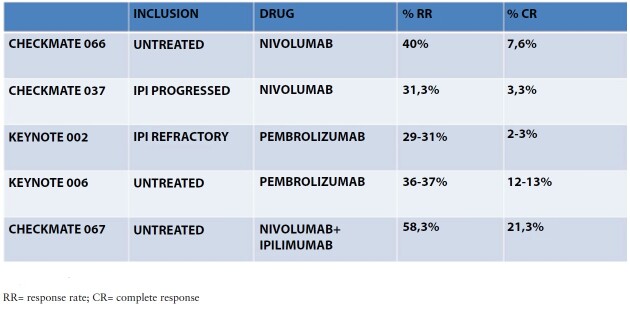
Clinical activity of immune check point inhibitors. RR= response rate; CR= complete response

**Figure 4 f4-dp11s1a164s:**
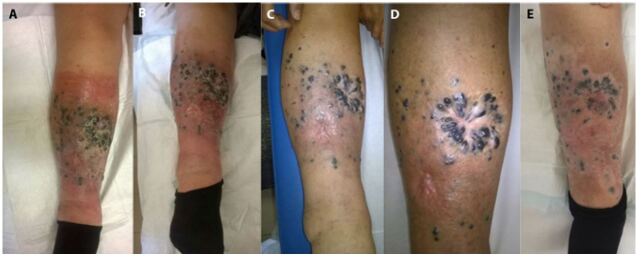
(A) Pattern of response following immune therapy in a patient with multiple in-transit skin metastasis localised in the lower limb. (B–D) Response developed during 1 year of treatment with induction of inflammation. (E) Immune activation around the skin metastases, towards complete clearance confirmed at histology with development of peri-lesional vitiligo.
